# Reducing variability in dual bolus cardiac MRI by using empirical contrast ratios

**DOI:** 10.1186/1532-429X-18-S1-W38

**Published:** 2016-01-27

**Authors:** Neil Chatterjee, Brandon C Benefield, Daniel C Lee, Timothy Carroll

**Affiliations:** 1 grid.465264.7Radiology, Northwestern University, Chicago, IL USA; 2 grid.465264.7Biomedical Engineering, Northwestern University, Chicago, IL USA; 3 grid.465264.7Cardiology, Northwestern University, Chicago, IL USA

## Background

Cardiac perfusion MRI enables quantification of myocardial blood flow in ml/min/g. However, signal intensity (SI) saturation of the arterial input function (AIF) leads to underestimation of the AIF and errors in perfusion quantification. Dual bolus experiments avoid this by using an unsaturated dilute bolus to measure the AIF and have been shown to be accurate against gold standard microsphere measurements. Despite the advantages associated with absolute quantification, dual bolus has seen limited adoption outside of a few large academic centers in part because it is difficult to implement. Set up requires either two injectors or a complex preloading scheme, both of which add time and complexity to a procedure that can be sensitive to experimental error. To calculate perfusion, the AIF from the dilute bolus is scaled to match the myocardial SI data from the full bolus using the ratio of contrast concentrations between the full and dilute boluses at the left ventricle. This is typically assumed to be the dilution ratio used to mix the dilute bolus, and any imprecision during mixing can translate into large errors in AIF height and thus perfusion calculations. Modifying the dual bolus technique so that it is more tolerant of experimental error might facilitate more widespread adoption outside of large research centers. Here we present a method for empirically determing contrast ratios that can be applied retropspectivley to dual bolus perfusion data to reduce experimental error.

## Methods

11 dual bolus (0.005 and 0.05 mmol/kg) firstpass perfusion studies were conducted on 5 dogs during rest and adenosine vasodilated stress on a 1.5 T clinical scanner. Myocardial blood flow was quantified by injected microspheres. Dual bolus correction was performed two separate ways: 1) using an assumed ratio of 10, 2) using least squares fitting to find the concentration ratio that minimized the differences between the dilute and full AIFs in the unsaturated tail region (Fig 1a) of the SI curve. Perfusion was calculated using a Tofts Kety two compartment model.

**Figure 1:**
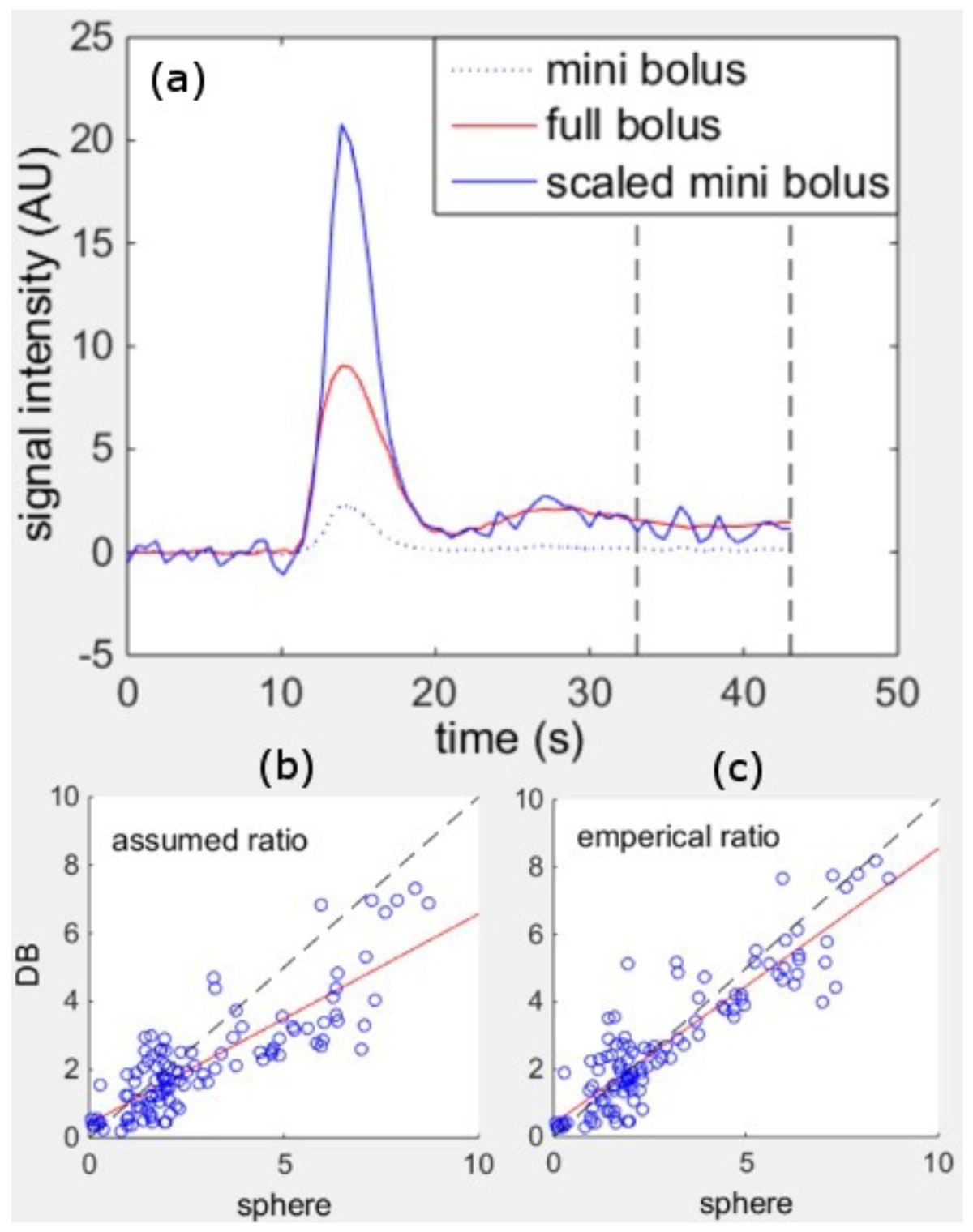
**a) The unsaturated portion of the full bolus AIF in a dual bolus experiment is used to empirically determine a contrast concentration ratio that best matches the dilute ("mini") bolus to the full bolus**. b,c) Using the emprically derived ratios improves MRI perfusion measurements against gold standard microsphere data Edit

## Results

Incorporating the tail correction to empirically determine contrast concentration ratios increased correlation against microsphere perfusion values from 84 to .90 (Fig 1b&c). The 95% confidence interval on the difference between correlation coefficients was [.035, .102], so this change was significant at p < .05. The empirically determined ratios of 8.5 +/ 1.5, were significantly different from the dilution ratio of 10 (p < .001).

## Conclusions

Using empirically determined contrast ratios improved MR perfusion correlation against gold standard reference values. Becaue this method does not assume a fixed dilution ratio between the two boluses, it is much more tolerant to any imprecision in experimental setup. This reduces the logistical burden on the staff carrying out a dual bolus experiment, which may facilitate more widespread adoption of this proven quantitative technique into busier clinical environments.

